# Arbuscular Mycorrhizal Fungus *Rhizophagus irregularis* Influences *Artemisia annua* Plant Parameters and Artemisinin Content under Different Soil Types and Cultivation Methods

**DOI:** 10.3390/microorganisms8060899

**Published:** 2020-06-15

**Authors:** Erzsébet Domokos, Béla Bíró-Janka, János Bálint, Katalin Molnár, Csaba Fazakas, László Jakab-Farkas, József Domokos, Csilla Albert, Gyöngyvér Mara, Adalbert Balog

**Affiliations:** 1 Department of Horticulture, Sapientia Hungarian University of Transylvania, Sighisoarei Street 1/C, 540485 Târgu Mureș, Romania; bela.biro86@gmail.com (B.B.-J.); balintjanos@ms.sapientia.ro (J.B.); molnarkati@ms.sapientia.ro (K.M.); fazakascs@ms.sapientia.ro (C.F.); 2 Department of Mechanical Engineering, Sapientia Hungarian University of Transylvania, Sighisoarei Street 1/C, 540485 Târgu Mureș, Romania; jakabfarkaslaszlo@gmail.com; 3 Department of Electrical Engineering, Sapientia Hungarian University of Transylvania, Sighisoarei Street 1/C, 540485 Târgu Mureș, Romania; domi@ms.sapientia.ro; 4 Department of Food Science, Sapientia Hungarian University of Transylvania, Piaţa Libertăţii 1, 530104 Miercurea Ciuc, Romania; albertcsilla@uni.sapientia.ro; 5 Department of Bioengineering, Sapientia Hungarian University of Transylvania, Piaţa Libertăţii 1, 530104 Miercurea Ciuc, Romania; maragyongyver@uni.sapientia.ro

**Keywords:** AMF, malaria, tropical disease control, artemisinin production, temperate zone, water supply, polyphenol oxidase, soil types

## Abstract

Artemisinin extracted from *Artemisia annua* has been used efficiently in malaria treatment since 2005. In this study, the variations in plant parameters (plant biomass, glandular trichome density, essential oil total chemical content, artemisinin production, and polyphenol oxidase (PPO) activity) were tested under different soil types (Luvisol, Gleysol, Anthrosol and sterile peat) and cultivation conditions (potted plants in semi-open field, and open field experiments) for plants inoculated with arbuscular mycorrhizal fungus (AMF) *Rizophagus irregularis*. Under semi-open field conditions, the AMF colonization of *A. annua* plant roots varied, and presented the highest percentage in Luvisol and sterile peat. The increase in the root colonization rate positively influenced some plant parameters (biomass, glandular trichome density, artemisinin concentration, essential oil quantity and composition), but no effects on PPO enzyme activity were detected. AMF fungus *R. irregularis* significantly increased the artemisinin content and essential oil yield of plants cultivated in Luvisol, Gleysol, Anthrosol and in peat. These soil types can offer appropriate conditions for *A. annua* cultivation and artemisinin production even on a smaller scale. Under open field conditions, low (about 5%) AMF colonization was observed. No differences in artemisin contents were detected, but essential oil yield significantly increased compared to control plants. AMF treatment increased beta-farnesene and germacrene D concentrations in *Artemisia* plants in the open field experiment.

## 1. Introduction

Malaria is the predominant infectious disease in developing nations, with 380 million people infected and between 1 and 3 million dying annually [1,2]. While substantial efforts are being made to control malaria, it is still estimated that it will remain among the most serious infectious diseases until 2030 [1]. Therefore, *Artemisia annua*, due to its active principles, offers the possibility to control malaria and is a real solution for other tropical diseases as well. Artemisinin acts 10 to 100 times more rapidly than other well-known medicines used for malaria treatment [3]. Altogether, the current knowledge about the effect of artemisinin suggests that this plant ‘can save Africa’ and even the whole tropical world [4]. While the production of different *A. annua* varieties has been concentrated in tropical climates, its cultivation under specific but cheap conditions is possible in the temperate zone as well. According to previous studies the artemisinin content of plants can be improved under specific growing conditions (by arbuscular mycorrhizal fungus (AMF) colonization) [5].

Only a few studies so far have tested the effect of AMF fungi on *A. annua* [6,7]. Some AMF species such as *Glomus macrocarpum* and *G. fasciculatum* had significant positive effect on the herbage production, dry weight and nutrient content (P, Zn and Fe) of the shoot. Essential oil and artemisinin content also increased in AMF-inoculated plants compared to controls [6]. Similarly, inocula of *G. mosseae* and *Bacillus subtilis* together resulted in an increase in plant biomass and artemisinin yield [7]. While many aspects of AMF symbiosis with plant roots are still unclear, recent studies have revealed that some AMF species such as *R. irregularis* secrete effector proteins that positively influence the mycorrhization process of fungi with plant roots. The same research has also demonstrated that *R. irregularis* secretes SP7 proteins, which interact with the pathogenesis-related transcription factor ERF19 in the plant nucleus, conferring high resistance against plant pathogens [8]. The over-expression of other proteins under AMF symbiosis than ERF transcription factors also conferred improved resistance against pathogen attack and several abiotic stresses [9,10].

The glandular hairs are responsible for artemisinin production and accumulation in *A. annua*. Several genes involved in artemisinin production expressed in the glandular hairs were revealed: *1-deoxy-D-xylulose-5-phosphate synthase* (*DXS1*) and *1-deoxy-D-xylulose-5-phosphate reductoisomerase* (*DXR1*) expressed in the subapical cells, and *2-C-Methyl-D-erythritol 2,4-cyclodiphosphate synthase* (*MECDP*), *amorpha-4,11-diene synthase* (*ADS*), *cytochrome P450 monooxygenase* (*CYP71AV1*) and *artemisinic aldehyde delta-11(13) reductase* (*DBR2*) localized in the glandular hair head [11]. Although it is known that inoculation of *A. annua* with specific AMF can increase the glandular hair density on the leaves in controlled conditions, and also that the morphology and density of glandular hairs influences secondary metabolite content in plants, information about these secretory structures are limited in the case of *A. annua* under AMF treatment and different growing conditions [12,13,14,15].

The variations in the chemical compounds in *A. annua* are also important parameters to study. Artemisinin and several plant metabolites obtained from *A. annua* could be effective in treating other parasitic diseases, several viral infections and various neoplasms [2,16,17]. Previous studies have demonstrated that the dominant phenolic compounds in *A. annua* plants are flavones, their glycosides (luteolin, luteolin-7-glucoside, apigenin), flavonols and their glycosides (kaempferol, quercetin, isoquercitrin, rutin, patuletin), coumarins (coumarin, 6,7-dimethoxy-coumarin) and phenolic acids (ferulic acid) [16,18]. These compounds are present in *A. annua* in small amounts, and therefore research into cultivation in order to enhance the quantity and quality of the active principles is of high interest [2].

In European agriculture, plant cultivation is practiced under greenhouse, semi-open field (potted plants in open field conditions with water and nutrient supply controlled) and complete open field conditions (still under water and nutrient supply controlled). Therefore, the possibility of these cultivation methods in the case of *A. annua* and especially the effect of these methods on plant parameters needs to be tested also in temperate climate conditions [19,20]. Moreover, no previous research has tested the effect of AMF on Artemisia plant parameters (plant biomass, trichome densities, artemisinin concentration and essential oil chemical profile) if different soil types are used as growing conditions. Furthermore, because the farming of *A. annua* is predominantly practiced under warm climatic conditions with limited water supply, the role of antioxidant enzymes such as polyphenol oxidase (PPO) in plants, when these are colonized by AMF and cultivated under different soil types and methods (semi-open field and open field), is completely unknown. The formation of glandular trichomes and trichome-borne metabolites has been related to jasmonic acid (JA) biosynthesis, which induces polyphenol oxidase activity in plants [21,22].

On the basis of this information, the main goals of the present study were to assess the variations in *A. annua* plant parameters (plant biomass, glandular trichome densities, essential oil total chemical content, artemisinin production and PPO enzyme activity) when plants are in mutualism with AMF R. irregularis under different soil types and cultivation conditions. The following hypotheses were formulated:

1. The AMF colonization of *A. annua* plant roots may vary under different soil types and cultivation methods (semi-open field and open field conditions);
2. The AMF colonization rate of A. annua plants may influence plant total biomass (root, stem and leaf), glandular trichome density, essential oil total chemical content, artemisinin concentration and polyphenol oxidase (PPO) enzyme activity under different soil types and cultivation methods;
3. Altogether, the effects of AMF colonization on plant parameters under different soil types and cultivation methods can be good indicators to consider the effectiveness of *A. annua* cultivation under temperate climate regimes.

## 2. Materials and Methods

### 2.1. Plant Used for Experiments, Growing Condition and AMF Inoculation

**Figure 1:**
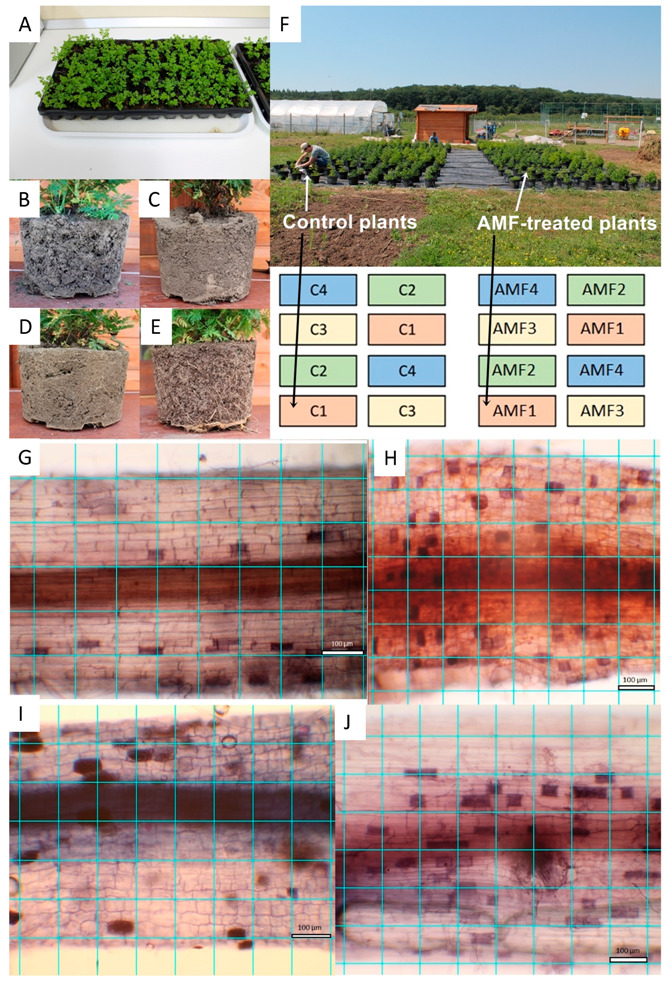
Experimental setup under semi-open field and open field conditions. *Artemisia annua* seedlings on germination tray with sterile peat before experiment start (**A**); soil structure of potted plants in Gleysol (**B**), Luvisol (**C**), Anthrosol (**D**) and sterile peat (**E**) during the experiment. Semi-open field experiment layout with 16 blocks shows control and arbuscular mycorrhizal fungus (AMF)-treated plants (**F**), different soil types are numbered as follows: 1-Gleysol, 2-Luvisol, 3-Anthrosol, 4-sterile peat. AMF colonization of *Artemisia* roots in various substrate types under microscopy analyses: Gleysol (**G**), Luvisol (**H**), Anthrosol (**I**) and sterile peat (**J**). One square represents 100 μm^2^.

*A. annua* A-3 (Anamed, Winnenden, Germany) seeds were used in the experiment. The seeds were sown in a climate chamber in March in multi-compartmental germination trays with commercial sterile peat (Blondy Romania SRL, Târgu Mureș, Romania) (Figure 1A). The climate chamber conditions were 22 °C, 40%, air humidity and 16 h light vs. 8 h dark photoperiod. For irrigation, 300 mL water/tray/day was used.

The seedlings with 5–6 leaves were inoculated with the AMF spores of R. irregularis (Italpollina SPA, Italy). From high concentration AMF powder (1400 spores/g), 0.5 g mycorrhizal inoculum was added to each germination tray after dissolving in 400 mL water. In the first 24 h after the treatment, the trays were not watered. Seedlings already AMF-inoculated and control plants without AMF were planted in pots of 20 L filled with commercial sterile peat and three soil subtypes: stagnic colluvic Gleysol, endostagnic argic Luvisol, and stagnic gleyic Anthrosol (Figure 1B–E). During the next 3 weeks, plants treated with AMF were kept separate from the control plants and all pots were transported into a greenhouse for adaptation to daylight [5].

The soils utilized in the experiment were collected from the surrounding habitats, being classified as the most common soils from the region. Soil samples were taken from the upper soil (ploughed layer 0–20 cm) from all three soil types, 15 cores for each representative bulk sample. The samples were submitted for analysis to the soil-testing laboratory of the Mureș County Office for Pedology and Agrochemistry. The total nitrogen amount (Kjeldahl method), available P (Egner-Riehm-Domingo method), available K (from the same extract as used for the P), soil pH (in H2O), humus content (Walkley-Black method), base saturation (Kappen method) and granulometrical classes (Pipette method) were determined. Danmuld, as a substrate for seeding (sphagnum peat moss) was used, with added nutrients (PGMix 1 kg/m^3^) and the following characteristics: dry matter content: 55–75 g/L, particle size: 0–20 mm, pH: 5.5, EC: 1.0–2.5.

Seedlings already AMF-inoculated were planted into open field soil, in the experimental garden of the university. The open field soil was stagnic vertic Luvisol, where the processes of humification, leaching, and clay formation are accompanied by the migration of the clay particles and moderate acidification. The soil horizons follow as shown: Ap 0–20 cm, Auw 20–40 cm, A/B 41–56 cm, Btwy1 56–70 cm, Btwy2 70–95 cm, B/C 95–120 cm, C 120. The soil-forming rock (bedrock) has a high montmorillonite and smectite clay content. It swells when wet—aeration conditions are diminished—and shrinks and cracks when dry, which could lead to root breakage. Due to the significant amount of clay in the soil, stagnant water persists and reducing conditions are formed during the year in the major part of the layers, especially in the clay accumulation zone—Bt horizon—resulting in large quantities of free iron and manganese oxides in the structural elements and on their surfaces by the clay-coating plaques (Table 1 and Table 2).

Here, we must emphasize that, during our previous experiments, we tried to add AMF spores to plant roots after sowing under open field conditions and after planting them in pots in the preferred soil. Our analysis demonstrated that there was no AMF colonization when plants were first sown and then AMF was added. Therefore, we applied AMF first under sterile conditions in peat, and after AMF colonization was detected (tested the first time under a microscope), plants were placed in their growing conditions. In this case, AMF colonization was standardized and AMF development under different soil types and cultivation methods was assessed further during the experiment.

### 2.2. Experiment Layout under Semi-Open Field and Open Field Conditions

All plants were placed in the university’s experimental garden in early June (46°31′18.18″ N, 24°35′53.78″ E). One 1000 m^2^ area, free of any other plants for around 50 m, was covered with a plastic wrap. This was done to avoid direct contact between the roots and soil. The semi-open field experiment consisted of 16 randomly placed blocks, each comprising 40 seedlings planted in pots containing the same substrate type (Figure 1F). The whole design was replicated for each soil type (sterile peat, Luvisol, Gleysol and Anthrosol) and, inside each block, 20 plants were selected as controls and 20 plants were previously treated with AMF. Inside each block, a 30 cm space was left between the nearest pots inside rows and 50 cm between pots and between rows. There was a 2 m distance between the blocks of control plants and those of the treated plants. The entire system was irrigated using an automatic drip source. During June, each plant received 400 mL of water/day, while, during July and August (when the temperature increased), plants received 800 mL/pot daily. Next, until the end of October (when plant materials were harvested) the water supply was reduced as in June. The standard and greenhouse conditions and recommended nutrient regime (N 250, P 50, K 250, Ca 100, Mg 30, Fe 2, Mn 0.3, B 0.3, Cu 0.1, Zn 0.26 ppm) were automatically provided bi-weekly for both AMF-treated and control plants at all soil types [5].

In the case of the open field experiment, plants were planted into Luvisol (in the experimental garden of the university) in early June, after AMF colonization. This was done because this soil type is the most frequent all around the area, representing about 85% of total soil types, and also because some plant parameters under semi-open field experiments were significantly improved under this soil type. The experiment consisted of four blocks (two for control plants with no AMF, and another two with AM-treated plants), each of the blocks comprising 80 plants (10 plants/row) (Figure S1 supplementary online materials). In each block, the spaces were 30 cm between the nearest plants inside rows and 50 cm between rows. There was a 10 m distance between the blocks of control plants and those of the treated plants. The plants were watered every day (except rainy days) by a sprinkler irrigation system. The same standard nutrients were provided automatically every 2nd week as previously described.

### 2.3. Testing AMF Development on Plant Roots after Colonization

In the case of the semi-open field experiment, the AMF colonization development under each of the soil types and cultivation conditions was evaluated on two randomly selected plants/treatments/blocks (32 plants) in the early vegetative period (one week after inoculation and placing in pots), and on the same sample size (32 plants) in the full vegetative period (eight week-old plants). From open field soil, for the same analysis, five randomly selected plants/treatments/blocks (80 plants) were collected in the two periods mentioned.

A sub-sample of about 150 root fragments (of 1 cm length) was chosen randomly from each plant. The staining of the root fragments was done in an acidic glycerol solution containing 0.05% trypan blue (Sigma-Aldrich, Romania) [23]. From each sub-sample, the first 10 root fragments were, again, randomly selected and each photographed under a light microscope (Ceti Topic-T, Belgium). Microscope pictures were taken at ×200 magnification using a Canon EOS 1100D, Taiwan camera. A ×200 magnification picture, which covered a segment of 0.882 mm length and 0.588 mm width, was taken of each fragment [5] (Figure 1G–J).

### 2.4. Glandular Hair Density Assessment in AMF and Control Plants

For the exact counting of foliar glandular hair numbers, the leaves were collected in July (four weeks after plants had been placed outside for the experiment). From leaves 2–4 (counting from the apical meristem), one of about 5 cm in length on each plant was removed for sampling. The leaf surface was analyzed by starting with a fragment from the leaf mounted on a scanning surface in the form of a 0.6 cm square (Figure S2A–E). The number of glandular hairs was counted with the multi-point tool, while the scanned leaf surface was measured with the polygon selection tool, in ImageJ version 1.51j8 (National Institute of Mental Health, Bethesda, MD, USA). Then, the leaf surface area was determined and the number of glandular hairs averaged and reported in mm^2^ [15]. In the case of the semi-open field experiment, three plants/treatments/blocks (two replicates from each soil type and a total of 24 AMF-treated and 24 control no-AMF plants as replicates) were used, while in the case of the open field soil experiment, 10 plants/blocks/treatments were selected. Scanning Electron Microscopy (SEM) was utilized (JEOL JSM-5200, Japan) to obtain the images of the leaf surface (upper epidermis). The following standard SEM conditions were used: secondary electrons at 20 mm working distance with a 1 to 5 kV accelerating voltage as function of the sample charge (Figure S2, online Supplementary Materials) [5].

### 2.5. Plant Biomass Measurements in AMF and Control Plants

In the case of the semi-open field experiment, 15 plants were selected randomly from each treatment and substrate type (60 AMF treated and 60 control no-AMF plants). Plants were collected in the full vegetative stage (in July). The weight of the root, stem and leaves of each plant were assessed after harvest using the following method: plant materials (root, stem and leaves) were separately washed and dried on blotting paper. Then, the fresh biomasses for roots, stems and leaves of each plant were individually measured. Finally, plant materials were dried for 3 weeks at 20 °C, and the dry biomass of roots, stems and leaves of each individual plant measured. By this time, 20 randomly selected plants/blocks from the open field experiments were also harvested, and the same assessment was made for fresh and dried plant materials.

### 2.6. Essential Oil Extraction and Total Chemical Content Analyses of AMF and Control Plants

Essential oils obtained from plants were subjected to gas chromatography–mass spectrometry (GC–MS) analysis. Essential oil was extracted from fresh leaves by hydro-distillation with a Clevenger apparatus. The oils were dried over anhydrous Na_2_SO_4_, frozen, and stored in sealed vials at −22 °C until GC–MS analysis [24]. The essential oil yield was given as the percentage determined based on measured oil volume (mL) obtained from 100 g of fresh leaves. All extractions were made with three replicates. The samples were analyzed with the Agilent 7890B GC–MS using the following standard conditions: fused silica HP-5 column, carrier gas He (1.1 mL/min), temperature program: 3 °C/min from 60 °C to 240 °C; the injection port temperature was 250 °C; the detector temperature was 280 °C. The ionization of the sample components was performed in EI mode (70 eV). The linear retention indices (RI) for all compounds were determined by the injection of the sample with a solution containing the homologous series of C_8_-C_26_ n-alkanes [25]. Essential oil constituents were analyzed and identified by comparing their retention times and mass spectra with data from the literature [25]. These data were extracted from the HP Chemstation computer library NBS75K.L, NIST/EPA/NIH (https://www.sisweb.com/manuals/nist05manual.pdf), Terpenoids Library (https://massfinder.com/wiki/Terpenoids_Library_List), and from the laboratory database [25].

### 2.7. Artemisinin Concentration Assessment in AMF and Control Plants

In the case of the semi-open field experiment, to determine the artemisinin content three plants/treatments/blocks (24 AMF treated and 24 control, no-AMF plants) were selected randomly. In the case of the open field soil experiment, 10 plants/treatments/blocks were used.

Pure artemisinin, used as standard, was purchased from Sigma-Aldrich (Germany). Plant (both with and without AMF) artemisinin contents were extracted as described previously [5]. First, the refluxing 100 g of dry leaves with hexane at 75 °C was carried out by leaving plant materials to dry for 1 h. Next, the hexane was evaporated under a vacuum and the samples reconstituted in 10 mL acetonitrile. The whole extract was filtered through 0.45 mm syringe filters. Standard HPLC analyses were performed using Agilent Infinity 1260 on Zorbax Eclipse Plus C18 (100 mm × 3.5 mm), column detection was conducted at 210 nm wavelength [5]. The acetonitrile:water 65:35% (*v/v*), was used as a mobile phase with a 0.6 mL/min flow rate [26]. The protocol by Lapkin et al. (2009) [27] was followed because it is a reliable laboratory analysis for the quantification of artemisinin in extracts. The calibration curve was constructed by plotting the peak area against the concentration (1.5325, 3.125, 6.25, 12.5, 25 mg/mL) of standard solutions [27]. The determination coefficient (R^2^) was 0.998.

The method has been validated as follows: Specificity and selectivity were studied for the examination of the presence of interfering endogenous components. A reference solution containing artemisinin was prepared along with a blank. The results indicate that the retention time of artemisinin was about 4.13 and none of the impurities interfered in its assay. Linearity was studied by preparing standard solutions at different concentration levels. The linearity range was found to be 0.5 to 25 mg/mL, R^2^ = 0.999. Accuracy was determined by assay and recovery studies of artemisinin. A known amount of standard artemisinin was added into the pre-analyzed sample and we subjected it to the proposed HPLC method. The study was carried out at three different concentration levels and the % recovery was found to be in the range of 94.72 to 103.56%. Intra- and inter-day variations were calculated to determine the precision of the method. The intra-day variation was determined for six concentration levels covering the analytic calibration range. The inter-day variation was determined through an analysis of these standard solutions on three consecutive days. The range for the accuracy was from 89.72 to 116.31%, while the coefficient of variance in both was less than 7.5%. The limit of detection (LOD) and quantification (LOQ) were calculated by the method based on the standard deviation (SD) of the response and the slope (S) of the calibration curve at levels approximating LOD = 3 (SD/S) and LOQ = 10 (SD/S), and found to be 10 µg/mL and 34 µg/mL, respectively.

### 2.8. Polyphenol Oxidase (PPO) Enzyme Extraction and Activity Assays

Leaf samples (500 mg sample/plant) used for enzyme analyses were collected in July, before flowering. In the case of the semi-open field experiment, three plants/treatments/blocks (24 AMF treated and 24 control, no-AMF plants) were used, while, in the open field soil experiment, eight plants/blocks were assessed. Homogenization methods described by Domokos et al., 2018 [15], were used. Plant leaves, after collection, were deposited at −20 °C until PPO enzyme extraction and activity assays. For PPO extraction, 200 mg of frozen *Artemisia* leaves were homogenized in 1 mL of QB buffer (100 mM KPO_4_ (pH 7.8), 1 mM EDTA, 1% Triton X-100, 10% glycerol, 1 mM DTT (added before use), distilled water), pH 7.8 using a FastPrep Instrument high-speed benchtop homogenizer (MP Biomedicals). The homogenate was centrifuged at 1000 g for 30 min at 4 °C, and the supernatant collected [5]. The protein concentration of the PPO enzyme extract was determined using the Bradford method [28]. The activity of PPO was determined at room temperature (20–25 °C) using a spectrophotometer. The detection conditions included a 495 nm wavelength and a reaction mixture (1 mL) containing 650 µL phosphate buffer (pH 7.5), 100 µL 3-methyl catechol, and 150 µL of crude protein extract (Bradford, 1976). Enzyme activity was assessed as described by Cavalcanti et al. (2004) [29]. According to this, one unit of PPO activity was defined as the amount of enzyme producing 1 mmol of quinone per minute. Specific activity was expressed in U/μg protein [29].

### 2.9. Data Analyses

For standardization, sample collections from plants were carried out randomly from each of the blocks and treatments, and the plants sampled (AMF, trichomes, biomass, artemisinin, PPO) were removed completely from the experiment, ensuring that the next samples were taken from intact plants.

The AMF colonization percentage and the exact number of glandular hairs from the upper epidermis of each of the leaves collected from plants under semi-open and open field conditions were determined by ImageJ Image Processing and Analysis in Java version 1.51j8 (National Institute of Mental Health, Bethesda, MD, United States) with the methods described in Domokos et al. (2018). On the images obtained from the microscope, a 0.1 mm^2^ area/point grid of lines was created. Next, all points were classified into the following groups: points with AMF colonization, points with vesicles, points with hyphae only, points with AMF material (one or more types), points with no AMF material and all examined points (Figure 1G,H). The AMF colonization (AC) and the vesicular colonization (VC) were counted by dividing the number of categories from the total number of examined points. The hyphal colonization (HC) was counted as the proportion of points with AMF material (Figure 1I,J) [15]. The data obtained were not distributed normally; therefore, to compare variables, the Kruskal–Wallis test was used, followed by the Mann–Whitney U test.

Glandular hairs from *Artemisia* leaves were counted exactly from each of the samples using the program ImageJ Image Processing and Analysis in Java version 1.51j8 (National Institute of Mental Health, Bethesda, MD, USA). The trichome data between AMF and control plants for each soil type under semi-open field conditions were compared using ANOVA and Tukey’s test; data under open field conditions were compared using a paired t-test. This was done because of the high differences in trichome numbers between semi-open field and open field conditions.

Plant biomass, artemisinin concentration, PPO data (both PPO content (U/mL) and its specific activity (U/ug)) were distributed normally (Shapiro–Wilk test) and homogeneously in variances (Levene’s test). Therefore, the analyses of the between-groups (treatments and control) data were done using one-way ANOVA followed by Tukey’s test. All statistics were performed on the R environment Community Analysis Package 4 [30]. Scores as predictor variables with F and p values were computed at 99.5% confidence intervals.

The effect of AMF on essential oil yield and composition was tested under different soil types and conditions (semi-open field and open field), considering all volatiles according to their relative proportion in the *Artemisia* plants. Because there were no differences in essential oil yield and composition between the same treatments (with and without AMF) inside the cultivation conditions (semi-open field and open field) (MANOVA *F* = 0.8, *p* < 0.54), comparisons were only made between treatments and cultivation types. This was done by first subjecting data about the relative amount of each chemical per plant/treatment (%) to a Principal Components Analyses (PCA). Next, the identification of the proportion of variation in each PCA axis was carried out and explained by AMF treatments and its absence for semi-open and open field conditions. The same method was used to test the effect of AMF treatment and its absence on the main chemical classes found in the *Artemisia* plants (monoterpene hydrocarbons, oxygenated monoterpenes and sesquiterpenes). In addition, PCA was used to test the effect of AMF colonization rate (without control in this case) as an environmental variable on the parameters assessed (glandular trichome numbers, root, stem and leaf biomass, PPO and artemisinin content). Before analyses, all data were first averaged, and log10 transformed, then PCA covariance analyses were computed using Community Analysis Package 4 in R [25].

## 3. Results

### 3.1. Growing Condition

The results obtained from soil chemistry analyses are displayed in Table 1. The particle size distributions in soils used in the semi-open field experiment are presented in Table 2. The pH of all three soil types was slightly acidic. They were formed on non-carbonated deluvial or colluvial sediments, with a high content of montmorillonite and illite clays. The presence of these minerals determined the high amount of available potassium cations in the soils. The available phosphorus content was poor in the Ap horizon (ploughed soil layer) of the Luvisol and Gleysol and moderate in the Anthrosol. The humus content was moderate in all the three soil types, the Luvisol was moderately and the other two soils deficiently supplied with nitrogen. The base saturation percentage was shown to be in accordance with the texture and the pH of the soils.

### 3.2. Root Colonization by AMF under Different Soil Types

**Figure 2:**
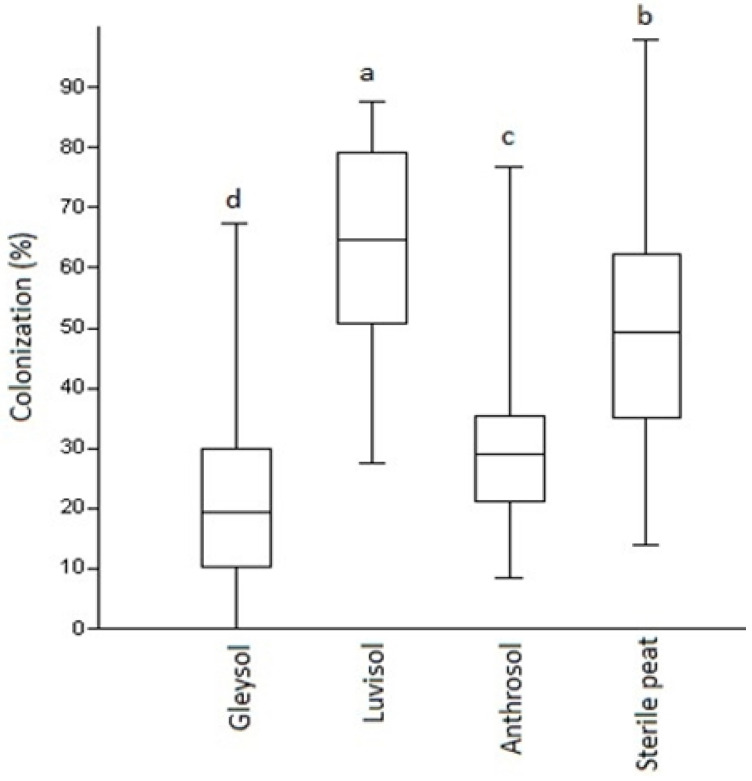
AMF colonization (%) of *Artemisia annua* roots under various substrate types. Kruskal–Wallis tests (non-parametric ANOVA) and Mann–Whitney U tests were used to compare variables (mean ± standard error). The different letters above the bar charts indicates statistically significant differences at *p* < 0.05 level (two replicates of five plants from each soil types as control and two replicates of five from each soil types as AMF treated, *n* = 80 plants). A sub-sample of about 150 root fragments (1 cm in length) was chosen randomly from each plant (80 × 150 and 12,000 as total) and average/treatment/plant determined. Bars represent standard errors.

While there were no differences in AMF colonization at the first assessment period (*U* = 0.9, *p* = 0.87), eight-week-old plants already presented significant differences in root colonization by AMF under the four substrate types used in the semi-open field experiment. The highest and most significant AMF colonization (up to 65%) has been detected in plants grown on Luvisol, followed by sterile peat (50%). The lowest AMF development was detected at the plant roots with Anthrosol (30%) followed by Gleysol (20%) (Figure 1G–J and Figure 2). In the open field, with Luvisol, the mean value for root colonization by AMF was very low, 5.098% (SD: 2.719, *n* = 20).

### 3.3. Glandular Hair Density Analysis

**Figure 3:**
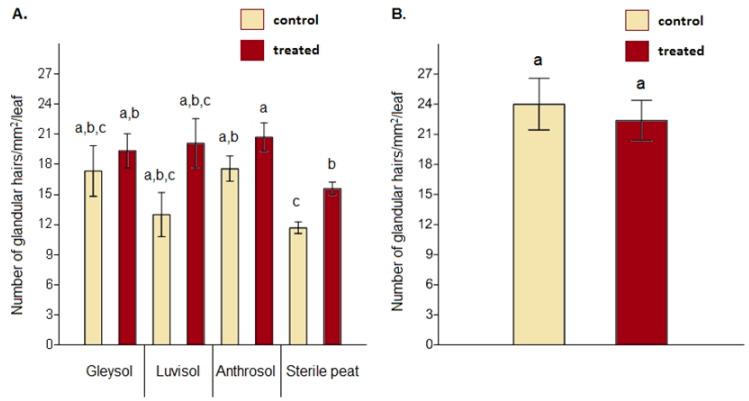
Glandular hair density on the upper leaf epidermis in the case of *Artemisia annua* under different growing conditions (**A**). Three plants/treatments/blocks (two replicates from each soil type and a total of 24 AMF treated and 24 control no-AMF plants as replicates) were analyzed. One-way ANOVA followed by Tukey’s test were used to compare variables (mean ± standard error). Glandular hair density between *Artemisia annua* control plants and AMF-treated plants cultivated under open field conditions (**B**). For this analysis, 10 plants/blocks/treatments were used. *F* and *t*-tests were used to compare variables (mean ± standard error). The different letters above the bar charts indicate statistically significant differences at *p* < 0.05 level.

When comparing glandular hair density between the control and AMF-treated plants, significant differences were obtained on sterile peat only (*F* = 2.35, *p* = 0.021) (Figure 3A, Table S1 supplementary online materials). Under open field conditions, no differences were detected between the controls and AMF-treated plants regarding glandular hair density (*F* = 1.648, *p* = 0.627) (Figure 3B).

### 3.4. Plant Biomass Analysis

**Figure 4:**
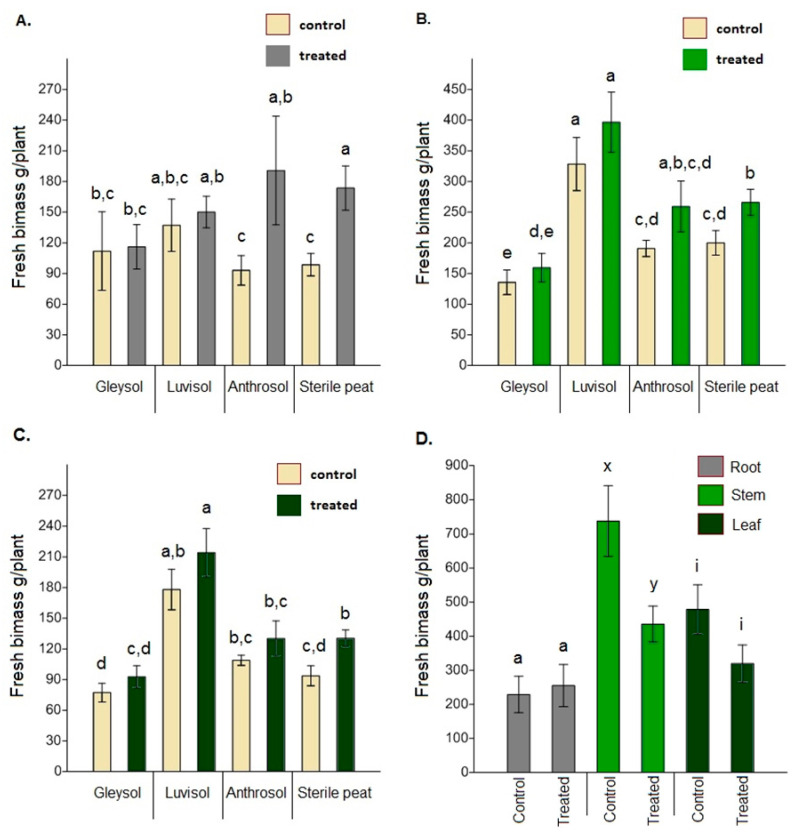
The fresh biomass of *Artemisia annua* vegetative organs under different growing conditions (**A**) root biomass, (**B**) stem biomass, (**C**) leaf biomass. Altogether 15 plants were selected randomly from each treatment and substrate type (60 AMF treated and 60 control no-AMF plants). One-way ANOVA followed by Tukey’s test were computed to compare variables (mean ± standard error). Fresh biomass of roots, stems and leaves under open field conditions (**D**). Altogether 20 randomly selected plants/blocks were collected and the same measurements were made using the same methods. *F* and *t*-tests were used to compare variables (mean ± standard error). The different letters above the bar charts indicates statistically significant differences at *p* < 0.05 level. Dry biomass data showed the same trend.

When comparing the fresh biomass of roots, stems and leaves between the control and AMF-treated plants, significant differences were only found for sterile peat (root: *F* = 3.934, *p* = 0.001; stem: *F* = 1.134, *p* = 0.037; leaf: *F* = 1.439, *p* = 0.008) (Figure 4A–C). Dried biomass data showed similar trends (root: *F* = 5.311, *p* = 0.014, stem: *F* = 2.146, *p* = 0.035; leaf: *F* = 2.58, *p* = 0.037). In the open field soil, control plants had a significantly higher fresh stem biomass than treated plants (*F* = 3.847, *p* = 0.012) (Figure 4D). The mean value for fresh leaf biomass was also higher (but not significantly) for control plants (*F* = 1.736, *p* = 0.09). Dried biomass data, again, showed similar trends (root: *F* = 1.342, *p* = 0.68; stem: *F* = 4.571, *p* = 0.062; leaf: *F* = 3.120, *p* = 0.077).

### 3.5. Essential Oil Yield and Composition

**Figure 5:**
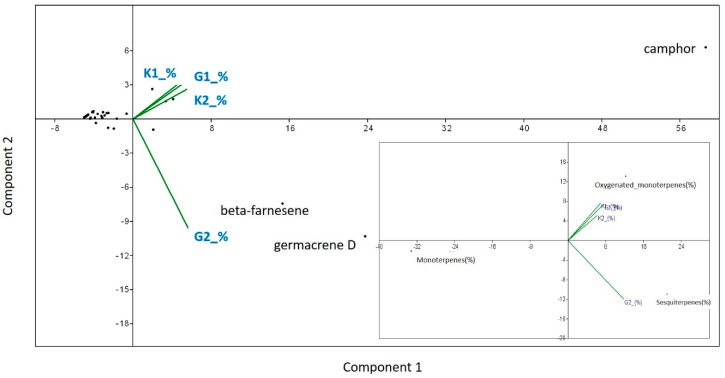
Principal Components Analyses (PCA) to detect the proportion of variation in each PCA axis on the relative amount of each chemical per plant/treatment (%). Variables were explained by AMF treatments and cultivation conditions (G1—semi-open field, G2—open field) and their absence (control) (K1-semi-open field, K2- open field). The same method was used to test the effect of AMF treatment and its absence on the main chemical classes found in the *Artemisia* plants (monoterpene hydrocarbons, oxygenated monoterpenes and sesquiterpenes).

In case of the semi-open field conditions, the essential oil obtained by hydro-distillation gave an average yield of 1.052% (SD: ± 0.052) for AMF-treated plants, and 0.552% (SD: ± 0.024) for control plants. The average yields of oil in open field conditions were 1.15% (SD: ± 0.1) for AMF-treated plants and 0.41% (SD: ± 0.017) for control plants. AMF-treated plants had significantly higher essential oil yields compared to the control plants both in semi-open field (*F* = 4.586, *p* = 0.033) and open field conditions (*F* = 32.972, *p* = 0.0001). Comparing AMF-treated plants yield from pots with those from open field soil, no significant differences were found (*F* = 3.664; *p* = 0.202), but control plants from semi-open field experiment gave an average yield significantly higher than control plants from open field soil (*F* = 1.961, *p* = 0.0001). The chemical profiles of the essential oils are reported in the Table S2 of supplementary online materials. Altogether, 36 compounds were identified, representing 91–95.23% of the total oil. In all the essential oils analyzed, the most abundant component was camphor (32.11–35.08%), except the AMF-treated plants from the open field experiment (G2), where, besides camphor (26.92%), beta-farnesene (16.85%) and germacrene D (23.52%) dominated the oil composition. The essential oils analyzed were rich in oxygenated monoterpenes (37.39–40.73%), except that of AMF-treated plants grown in open field soil (G2), where the most abundant components were sesquiterpenes (52.77%) (Table S2 of supplementary online materials). PCA also revealed that AMF treatment increased beta-farnesene and germacrene D concentrations in *Artemisia* plants in the open field experiment (G2). Overall, a positive effect of AMF on sesquiterpenes under open field conditions can be detected (Figure 5).

### 3.6. Artemisinin Concentration and PPO Enzyme Activity

The artemisinin concentration increased significantly in all substrates under AMF colonization. No differences, however, in artemisinin concentrations were detected between the control and AMF treatment under open field conditions (Table 3). No polyphenol oxidase content (U/mL), nor its specific activity (U/ug) were influenced by the AMF treatment (Table 4).

**Figure 6:**
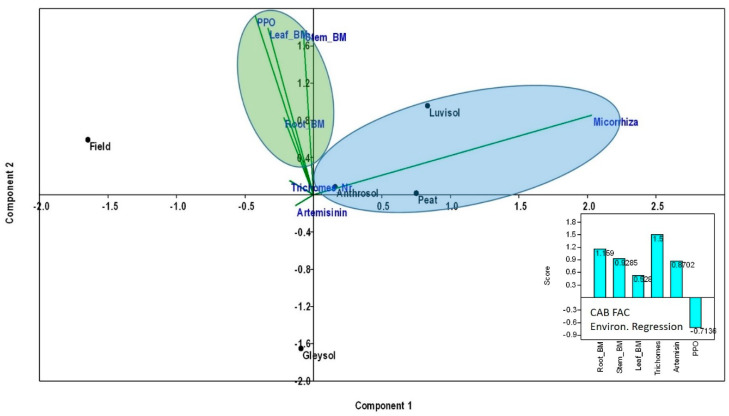
Principal Components Analyses (PCA) to test the effect of AMF colonization rates (without control in this case) as environmental variables on the assessed plant parameters (glandular trichome numbers, root, stem and leaf biomass, PPO and artemisinin content). Before analyses, all data were averaged and log10 transformed. PCA and CAB factorial environmental regression scores were computed using Community Analysis Package 4 in R.

When using PCA to test the effect of AMF treatments (without control in this case) as environmental variables on the assessed parameters (glandular trichome numbers, root, stem and leaf biomass, PPO and artemisinin content), a positive effect on plant parameters in Anthrosol, peat and Luvisol can be detected (Figure 6). The environmental regression equation indicates the positive effect of AMF on trichome numbers, followed by root and stem biomass and then the artemisinin content. No (or even a negative) effect of AMF on PPO concentrations and activity was detected (Figure 6).

## 4. Discussion

This is the first comparative study on the effect of the AMF fungus *Rhizophagus irregularis* on *Artemisia annua* under different growing conditions and cultivation systems (sterile peat, three soil types and open field soil). It can be detected that AMF colonization of *A. annua* plant roots varies under different soil types (confirming our first hypothesis). Significant differences in root colonization by *R. irregularis* were observed in the four growing conditions used in the pot experiment. Colonization presented the highest percentage in Luvisol and sterile peat.

The AMF colonization rate of *A. annua* plants influenced some plant parameters (biomass, glandular trichome density, artemisinin concentration and essential oil total chemical content), but no effect on PPO enzyme activity was detected (our second hypothesis was partially confirmed) under the semi-open field experiment. In more detail, it can be detected that *R. irregularis* has a positive effect on *A. annua* biomass, foliar glandular hair density and artemisinin content when plants are cultivated in Luvisol and in sterile peat. AMF fungus *R. irregularis* increased the artemisinin content of *A. annua* cultivated under all soil types when plants were potted. The effects of *R. irregularis* on the studied parameters (biomass, foliar glandular hair density, artemisinin content, PPO content and activity) in open field soil conditions were almost unobservable. Low (about 5%) AMF colonization was detected under open field conditions and, even if stem biomass and glandular hair density decreased in AMF plants compared with the control under these conditions, there were no differences in artemisinin content between AMF and control plants.

It is known that plant–host colonization depends not only on the chemical properties and resource availability of the soil [29], but also on the native species present in the soil [31]. Usually the positive effect of AMF on plants is more visible when root colonization is more intense and no or fewer other microorganisms are present. However, when the environmental conditions (water, nutrient availability) are appropriate for the plant host, the effect of AMF tends to have less importance in the growth and development processes of the host [29,30]. These findings could explain the results we obtained for plants in semi-open field experiments, where (probably) optimal nutrient and water supply and a less deleterious effect of indigenous microorganisms on AMF development occurred compared with open field conditions.

The accumulation of artemisinin in AMF-treated plants is enhanced both by the increase in foliar glandular hair density [5] and the upregulation of the genes involved in artemisinin biosynthesis [32]. According to our results, foliar glandular hair density was higher in AMF-treated plants compared to control plants under all growing conditions, but these differences were significant only in sterile peat. Even so, significant increases in artemisinin content were obtained on all soil types under AMF treatment. AMF inoculation had no effect on PPO content and specific activity under the different growing conditions. In an earlier study, it was found that AMF-treated *Artemisia* plants cultivated in sterile peat have a significantly higher guaiacol peroxidase activity than control plants [5]. In the present study, probably because of the stable water and nutrient supply, there were no significant stress effects on plants. Therefore, we shall carry out further research to test the effect of different water supply levels on *Artemisia* plant parameters under different growing conditions.

AMF-treated plants had significantly higher essential oil yields compared to control plants both in semi-open field (with an average of 90%) and open field conditions (with an average of 180%). The same results were obtained in [33], where treatment with *Glomus fasciculatum* increased the essential oil concentration, by 66% compared to control plants. In this study, a low positive correlation or no correlation was found between artemisinin and essential oil content in three *A. annua* accessions [6]. Under open field conditions, AMF-treatment also had a positive effect on the sesquiterpene content (especially beta-fernasene and germacrene-D) of the essential oil.

When analyzing the plants inoculated with AMF, it was found that the colonization percentage in roots has a positive effect on glandular hair density, followed by root and stem biomass, and then the artemisinin content. To make clear correlations between the presence of *R. irregularis* in the different substrates and the parameters studied, the substrate-associated microorganisms must be genetically determined. Further analyses are necessary regarding the quantitative and qualitative characterization of essential oil from the plants under the effect of AMF inoculation and different growing conditions. In addition, alongside these analyses, competition experiments with *R. irregularis* and other soil-inhabiting microorganisms would be of high interest.

Overall, it can be concluded that the effects of AMF colonization on plant parameters under different soil types and cultivation methods can be good indicators to consider the effectiveness of *A. annua* cultivation under temperate climate conditions. Luvisol, Anthrosol, Gleysol and sterile peat can offer appropriate conditions for *A. annua* cultivation and artemisinin production, even on a smaller scale and under semi-open field conditions. Artemisinin cultivation in small-scale culture is currently under investigation by our research group. The effectiveness of cultivation, water and nutrients in plants under open field conditions in temperate regions, as well as the artemisinin content and a total economic evaluation of cultivation, must be established. New assessments of these issues are currently underway.

## Figures and Tables

**Table 1:** Nutrient content, and accessibility of the soil types used in semi-open and open field experiment.

Soil Type	Hor.	Sampling Depth	pH	Available P (ppm)	Available K (ppm)	Humus %	Total N %	Base (sum) meq/100 g	Acidity (H+) meq/100 g	Total (T Value) meq/100 g	% BS
Gleysol	Ap	0–20	6.14	38	244	2.57	0.115	15.12	6.08	21.20	71.32
Luvisol	Ap	0–20	6.38	56	408	3.26	0.143	18.00	5.12	23.12	77.85
Anthrosol	Ap	0–20	6.11	82	334	2.18	0.102	17.28	6.40	23.68	72.97
Field-Luvisol			6.47	58	354	2.64	0.110	17.75	5.10	23.10	75.46

**Table 2:** Particle size distribution in soils used in semi-open and open field experiment.

Soil Type	Coarse Sand (2.0–0.2 mm)	Fine Sand (0.2–0.02 mm)	Silt (0.02–0.002 mm)	Clay (<0.002 mm)	Textural Class
Gleysol	15.8	24.0	21.8	38.4	Clay loam
Luvisol	8.6	21.6	29.2	40.5	Clay
Anthrosol	15.6	17.7	25	41.7	Clay
Field-Luvisol	9	22	29	40.1	Clay

**Table 3:** Comparison of artemisinin content (mg/g leaf) between *Artemisia annua* control plants and AMF-treated plants. ANOVA followed by Tukey’s test were used to compare variables. Statistically significant differences at *p* > 0.05 were considered.

	Artemisinin Content (mg/g Leaf)	
Substrate Types	Control Plants	AMF Plants
Gleysol	Mean: 3.65 *F* = 2.55	Mean: 4.89 *p* = 0.005
Luvisol	Mean: 4.12 *F*= 3.90	Mean: 4.24 *p* = 0.002
Anthrosol	Mean: 3.61 *F* = 3.99	Mean: 4.60 *p* = 0.002
Sterile peat	Mean: 3.55 *F* = 3.99	Mean: 4.83 *p* = 0.006
Open field conditions	Mean: 4.75 *F* = 1.78	Mean: 5.31 *p* = 0.06

**Table 4:** Comparison of polyphenol oxidase (PPO) content (U/mL) and its specific activity (U/ug) between *Artemisia annua* control plants and AMF-treated plants. ANOVA followed by Tukey’s test were used to compare variables. Statistically significant differences at *p* > 0.05 were considered. In the case of the semi-open field experiment three plants/treatments/blocks (24 AMF treated and 24 control, no-AMF plants) were used, while, in the case of the open field soil experiment, eight plants/blocks were assessed.

	Polyphenol Oxidase (PPO) Content (U/mL)	
Substrate Types	Control Plants	AMF Plants
Gleysol	Mean: 53.80 *F* = 6.92	Mean: 47.23 *p* = 0.43
Luvisol	Mean: 71.70 *F* = 3.07	Mean: 101.17 *p* = 0.62
Anthrosol	Mean: 54.92 *F* = 4.9	Mean: 108.53 *p* = 0.336
Sterile peat	Mean: 76.08 *F* = 5.39	Mean: 80.74 *p* = 0.89
Open field conditions	Mean: 105.67 *F* = 1.059	Mean: 162.16 *p* = 0.12
	Polyphenol oxidase (PPO) specific activity (U/ug)	
Gleysol	Mean: 0.10 *F* = 1.42	Mean: 0.18 *p* = 0.16
Luvisol	Mean: 0.27 *F* = 6.83	Mean: 0.17 *p* = 0.76
Anthrosol	Mean: 0.09 *F* = 2.32	Mean: 0.11 *p* = 0.69
Sterile peat	Mean: 0.15 *F* = 1.81	Mean: 0.28 *p* = 0.31
Open field conditions	Mean: 0.234 *F* = 16.65	Mean: 0.114 *p* = 0.29

## Supplementary Materials

The following are available online at https://www.mdpi.com/2076-2607/8/6/899/s1, Figure S1: *Artemisia annua* planted in open field stagnic vertic Luvisol: control plants (a) and AMF-treated plants (b), Figure S2: *Artemisia annua* plants glandular hair density on upper leaf epidermis under different growing conditions: Gleysol (a), Luvisol (b), Anthrosol (c), sterile peat (d) and open field of stagnic vertic Luvisol soil (e) (scanning electron microscope JEOL JSM-5200, Photos by László Jakab-Farkas), Table S1: The measured leaf surfaces (mean±SD), the number of glandular hairs counted (mean ± SD) on the measured leaf surfaces, and the density of glandular hairs/mm2 (mean ± SD) in case of control and AMF treated Artemisia plants, Table S2: The essential oil profile of the Artemisia plants under AMF treatment and without AMF (control) under semi-field (K1–control, G1-AMF treated) and open field conditions (K2–control, G2-AMF treated).

## Abbreviations

AMF	Arbuscular mycorrhizal fungus
PPO	Polyphenol oxidase
Commercial sterile peat	Sterile peat
Endostagnic argic luvisol	Luvisol
Stagnic colluvic gleysol	Gleysol
Stagnic gleyic anthrosol	Anthrosol

## References

[1] Mathers C.D., Loncar D. (2006). Projections of Global Mortality and Burden of Disease from 2002 to 2030. PLoS Med..

[2] Weathers P.J., Arsenault P.R., Covello P.S., McMickle A., Teoh K.H., Reed D.W. (2011). Artemisinin production in *Artemisia annua*: Studies in planta and results of a novel delivery method for treating malaria and other neglected diseases. Phytochem. Rev..

[3] Bridgford J.L., Xie S.C., Cobbold S.A., Pasaje C.F.A., Herrmann S., Yang T., Gillett D.L., Dick L.R., Ralph S.A., Dogovski C. (2018). Artemisinin kills malaria parasites by damaging proteins and inhibiting the proteasome. Nat. Commun..

[4] Zhu L., Tripathi J., Rocamora F.M., Miotto O., van der Pluijm R., Voss T.S., Mok S., Kwiatkowski D.P., Nosten F., Day N.P.J. (2018). The origins of malaria artemisinin resistance defined by a genetic and transcriptomic background. Nat. Commun..

[5] Domokos E., Jakab-Farkas L., Darkó B., Bíró-Janka B., Mara G., Albert C., Balog A. (2018). Increase in *Artemisia annua* Plant Biomass Artemisinin Content and Guaiacol Peroxidase Activity Using the Arbuscular Mycorrhizal Fungus *Rhizophagus irregularis*. Front. Plant. Sci..

[6] Chaudhary V., Kapoor R., Bhatnagar A.K. (2008). Effectiveness of two arbuscular mycorrhizal fungi on concentrations of essential oil and artemisinin in three accessions of *Artemisia annua* L.. Appl. Soil Ecol..

[7] Awasthi A., Bharti N., Nair P., Singh R., Shukla A.K., Gupta M.M., Darokar M.P., Kalra A. (2011). Synergistic effect of Glomus mosseae and nitrogen fixing *Bacillus subtilis* strain Daz26 on artemisinin content in *Artemisia annua* L.. Appl. Soil Ecol..

[8] Kloppholz S., Kuhn H., Requena N. (2011). A Secreted Fungal Effector of Glomus intraradices Promotes Symbiotic Biotrophy. Curr. Biol..

[9] Lee J.-H., Hong J.-P., Oh S.-K., Lee S., Choi D., Kim W. (2004). The ethylene-responsive factor like protein 1 (CaERFLP1) of hot pepper (*Capsicum annuum* L.) interacts in vitro with both GCC and DRE/CRT sequences with different binding affinities: Possible biological roles of CaERFLP1 in response to pathogen infection and high salinity conditions in transgenic tobacco plants. Plant. Mol. Biol..

[10] Zhang G., Chen M., Li L., Xu Z., Chen X., Guo J., Ma Y. (2009). Overexpression of the soybean GmERF3 gene, an AP2/ERF type transcription factor for increased tolerances to salt, drought, and diseases in transgenic tobacco. J. Exp. Bot.

[11] Xiao L., Tan H., Zhang L. (2016). *Artemisia annua* glandular secretory trichomes: The biofactory of antimalarial agent artemisinin. Sci. Bull..

[12] Kapoor R., Chaudhary V., Bhatnagar A.K. (2007). Effects of arbuscular mycorrhiza and phosphorus application on artemisinin concentration in *Artemisia annua* L.. Mycorrhiza.

[13] Mandal S., Upadhyay S., Wajid S., Ram M., Jain D.C., Singh V.P., Abdin M.Z., Kapoor R. (2015). Arbuscular mycorrhiza increase artemisinin accumulation in *Artemisia annua* by higher expression of key biosynthesis genes via enhanced jasmonic acid levels. Mycorrhiza.

[14] Suberu J., Song L., Slade S., Sullivan N., Barker G., Lapkin A.A. (2013). A rapid method for the determination of artemisinin and its biosynthetic precursors in *Artemisia annua* L. crude extracts. J. Pharm. Biomed. Anal..

[15] Stefanache C.P., Bujor O., Necula R., Danila D., Ciocârlan N. Phenolic Content of *Artemisia Annua* L. from Natural Habitats in Republic of Moldova—ProQuest. https://search.proquest.com/openview/0a6465eddb891c1456215087ced38ed3/1?pq-origsite=gscholar&cbl=676306.

[16] Ivanescu B., Vlase L., Corciova A., Lazar M.I. (2010). HPLC-DAD-MS study of polyphenols from *Artemisia absinthium*, *A. annua* and *A. vulgaris*. Chem. Nat. Compd..

[17] Jelodar N.B., Bhatt A., Mohamed K., Keng C.L. (2014). New cultivation approaches of *Artemisia annua* L. for a sustainable production of the antimalarial drug artemisinin. JMPR.

[18] Pulice G., Pelaz S., Matías-Hernández L. (2016). Molecular Farming in *Artemisia annua*, a Promising Approach to Improve Anti-malarial Drug Production. Front. Plant Sci..

[19] Bosch M., Berger S., Schaller A., Stintzi A. (2014). Jasmonate-dependent induction of polyphenol oxidase activity in tomato foliage is important for defense against Spodoptera exigua but not against Manduca sexta. BMC Plant Biol..

[20] Chen G., Klinkhamer P.G.L., Escobar-Bravo R., Leiss K.A. (2018). Type VI glandular trichome density and their derived volatiles are differently induced by jasmonic acid in developing and fully developed tomato leaves: Implications for thrips resistance. Plant Sci..

[21] Koske R.E., Gemma J.N. (1989). A modified procedure for staining roots to detect VA mycorrhizas. Mycol. Res..

[22] Adams D. (2007). Identification of Essential Oil Components by Gas. Chromatography/Mass Spectrometry.

[23] Bradford M.M. (1976). A rapid and sensitive method for the quantitation of microgram quantities of protein utilizing the principle of protein-dye binding. Anal. Biochem..

[24] Cavalcanti F.R., Oliveira J.T.A., Martins-Miranda A.S., Viégas R.A., Silveira J.A.G. (2004). Superoxide dismutase, catalase and peroxidase activities do not confer protection against oxidative damage in salt-stressed cowpea leaves. New Phytol..

[25] R Core Team (2013). R: A Language and Environment for Statistical Computing.

[26] Sendek A., Karakoç C., Wagg C., Domínguez-Begines J., do Couto G.M., van der Heijden M.G.A., Naz A.A., Lochner A., Chatzinotas A., Klotz S. (2019). Drought modulates interactions between arbuscular mycorrhizal fungal diversity and barley genotype diversity. Sci. Rep..

[27] Engelmoer D.J.P., Behm J.E., Toby Kiers E. (2014). Intense competition between arbuscular mycorrhizal mutualists in an in vitro root microbiome negatively affects total fungal abundance. Mol. Ecol..

[28] Gosling P., Mead A., Proctor M., Hammond J.P., Bending G.D. (2013). Contrasting arbuscular mycorrhizal communities colonizing different host plants show a similar response to a soil phosphorus concentration gradient. New Phytol..

[29] Donato R. (2015). Antibacterial activity of tuscan *artemisia annua* essential oil and its major components against some foodborne pathogens. LWT Food Sci. Technol..

[30] Lapkin A.A., Walker A., Sullivan N., Khambay B., Mlambo B., Chemat S. (2009). Development of HPLC analytical protocols for quantification of artemisinin in biomass and extracts. J. Pharm. Biomed. Anal..

[31] Martín-Robles N., Lehmann A., Seco E., Aroca R., Rillig M.C., Milla R. (2018). Impacts of domestication on the arbuscular mycorrhizal symbiosis of 27 crop species. New Phytol..

[32] https://www.sisweb.com/manuals/nist05manual.pdf.

[33] https://massfinder.com/wiki/Terpenoids_Library_List.

