# Supplementary material for: Arbuscular Mycorrhizal Fungus Rhizophagus irregularis Influences Artemisia annua Plant Parameters and Artemisinin Content under Different Soil Types and Cultivation Methods
Source: Microorganisms. 2020 Jun 15;8(6):899. doi: 10.3390/microorganisms8060899 (PMC7356791; doi:10.3390/microorganisms8060899)
Supplement: Supplementary file 1 [file microorganisms-08-00899-s001.pdf]

Supplementary online materials

Figure S1. *Artemisia annua* planted in open field stagnic vertic Luvisol: control plants (a) and AMF-treated plants (b).

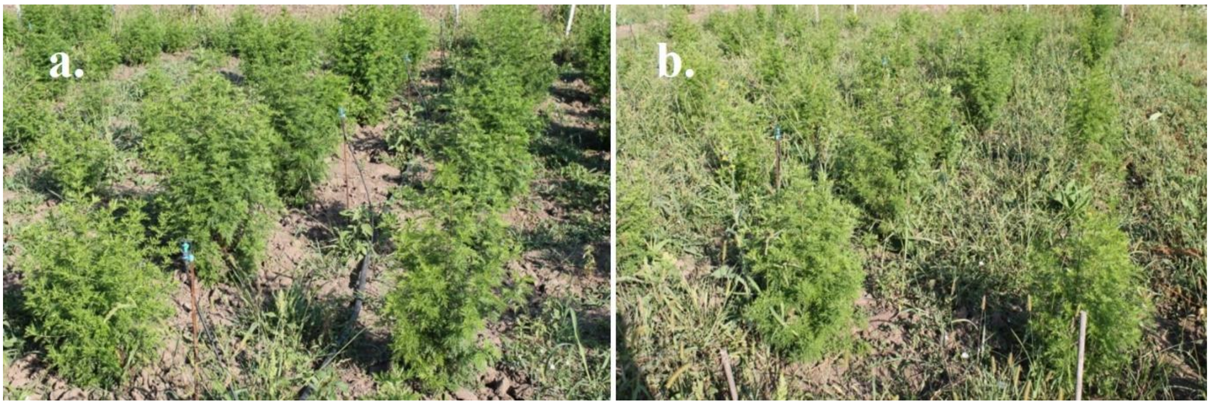

Figure S2. *Artemisia annua* plants glandular hair density on upper leaf epidermis under different growing conditions: Gleysol (a), Luvisol (b), Anthrosol (c), sterile peat (d) and open field of stagnic vertic Luvisol soil (e) (scanning electron microscope JEOL JSM-5200, Photos by László Jakab-Farkas).

a. 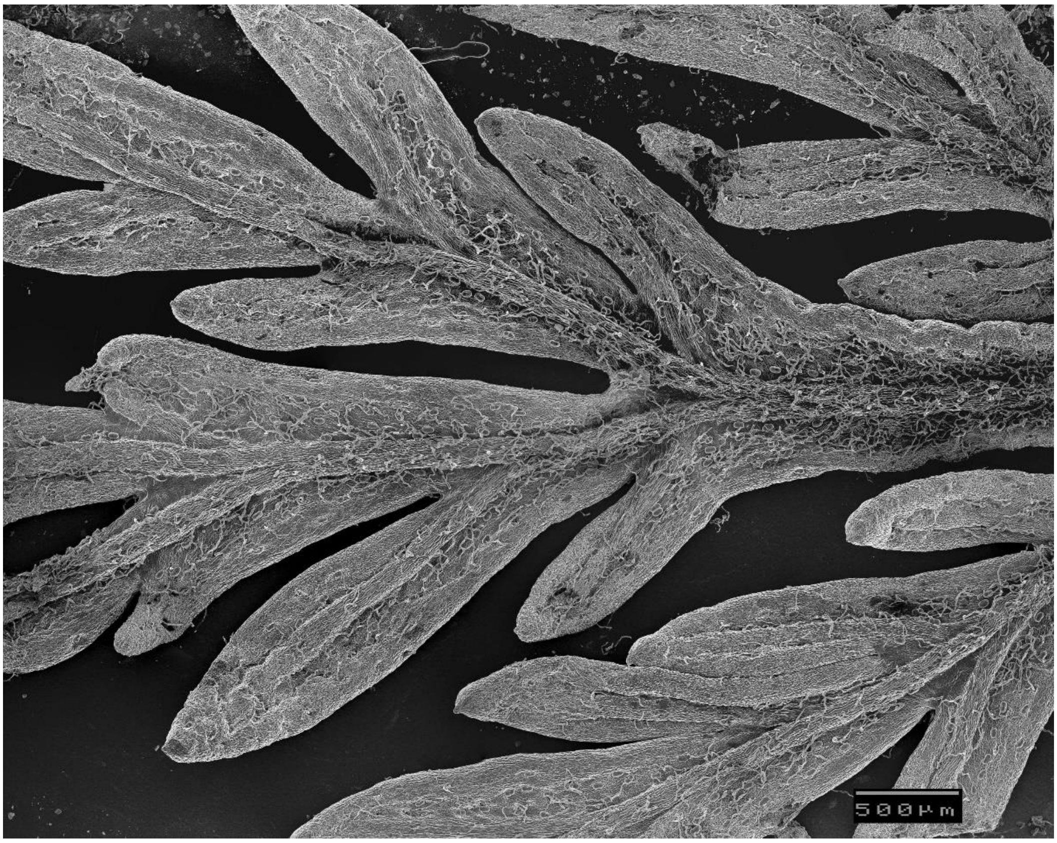

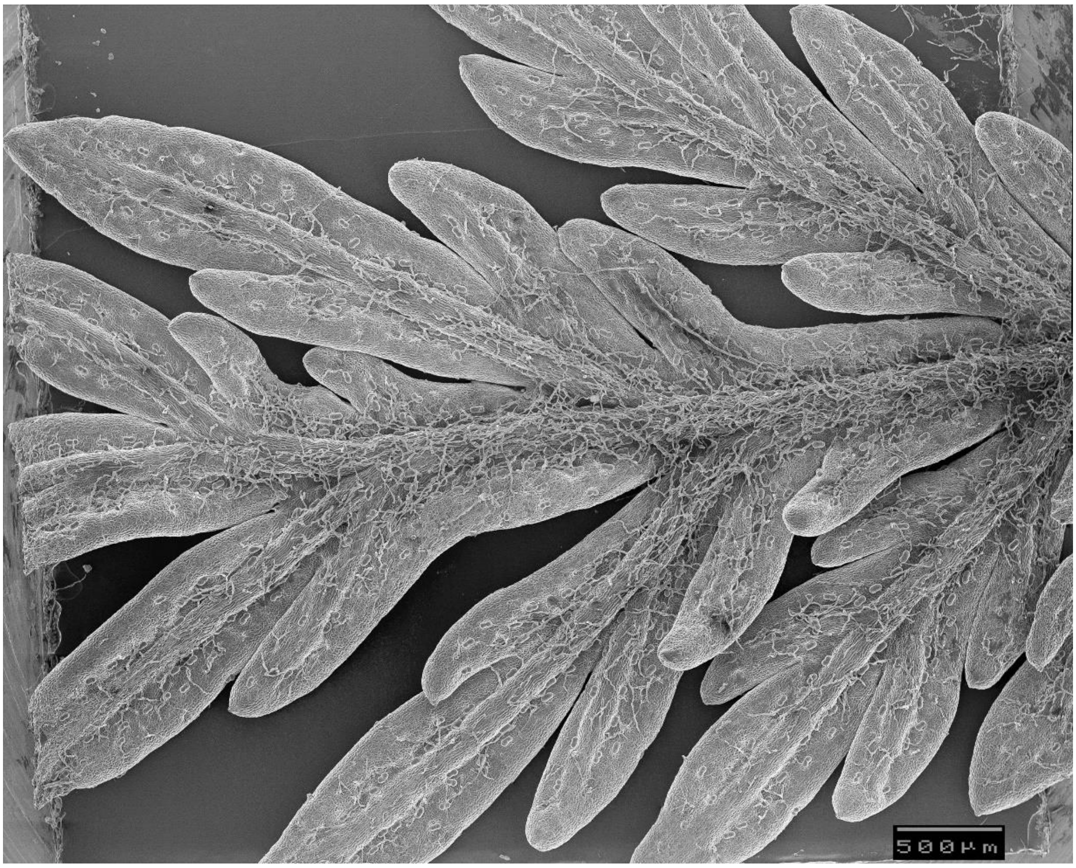

b.

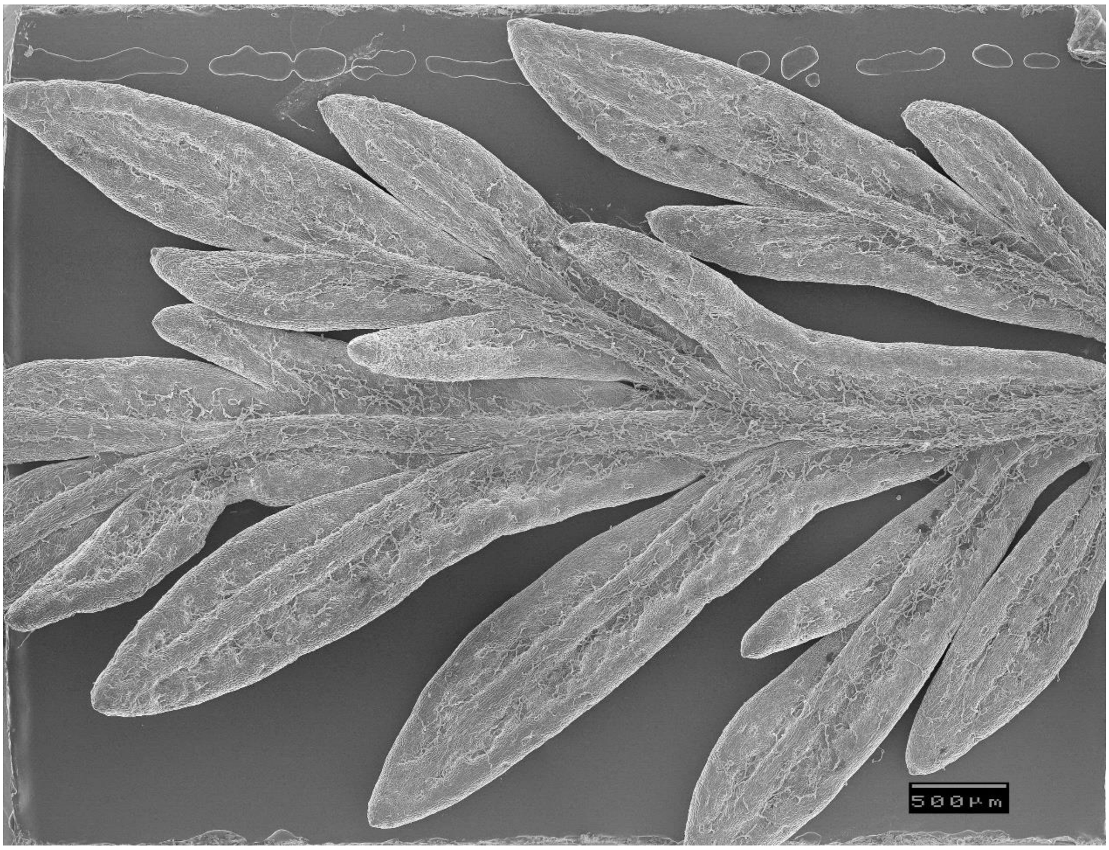

c.

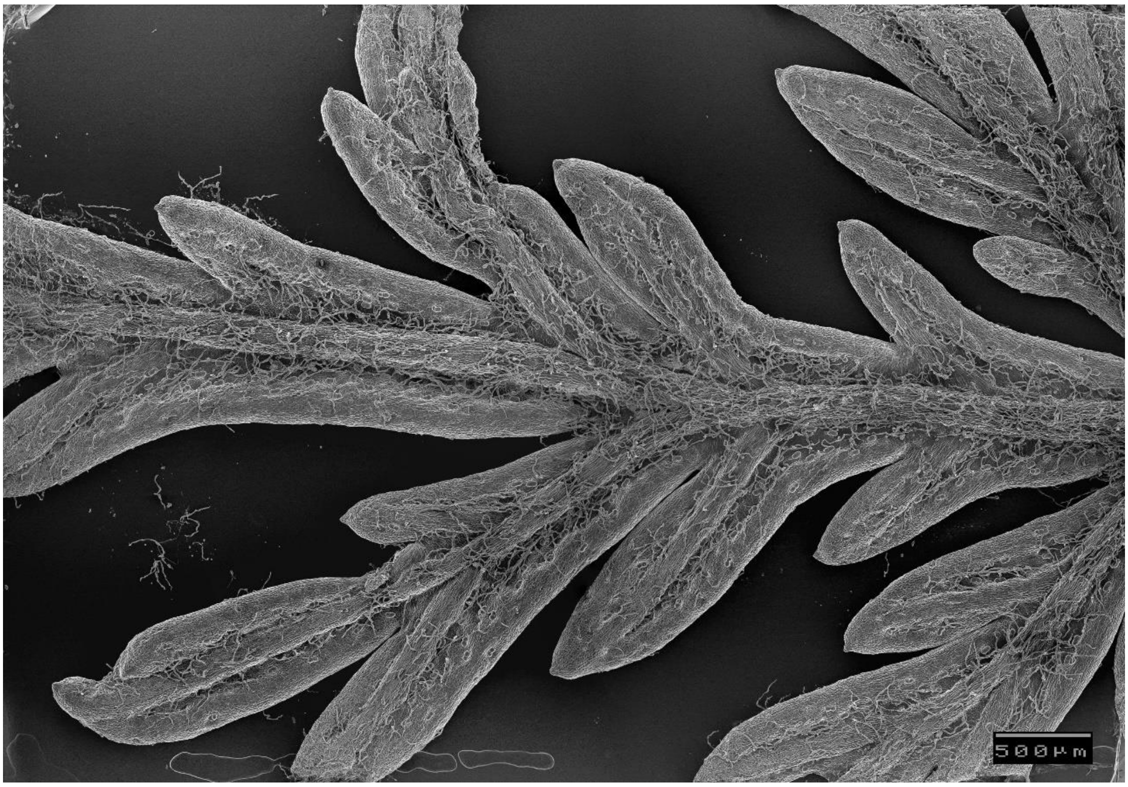

d.

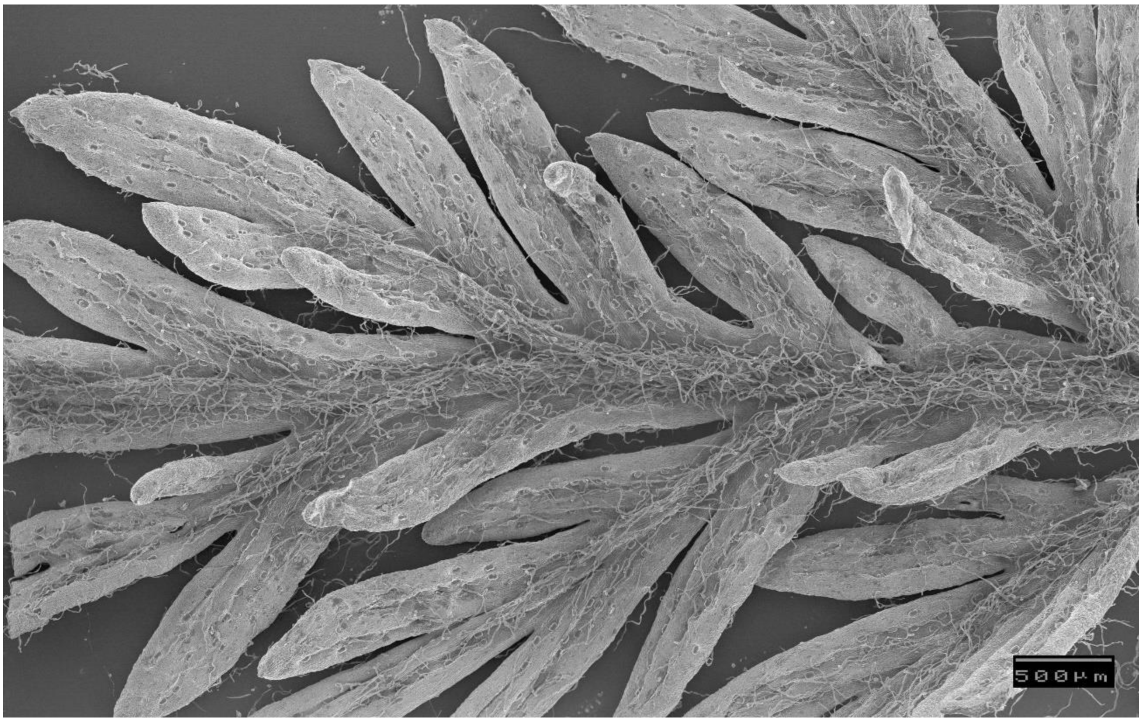

e.

Table S1. The measured leaf surfaces (mean±SD), the number of glandular hairs counted (mean±SD) on the measured leaf surfaces, and the density of glandular hairs / mm^2 (mean±SD) in case of control and AMF treated *Artemisia* plants.

	Measured leaf surface (mm^2)		Number of hairs on the measured leaf surface		Glandular hair density / mm^2	
Semi-open field						
Control plants	Mean	±SD	Mean	±SD	Mean	±SD
Gleysol	186.908	14.842	3282.400	1417.776	17.335	6.920
Luvisol	201.848	13.948	2574.000	1085.689	12.992	6.031
Anthrosol	187.123	18.193	3264.800	584.109	17.578	3.457
Sterile peat	173.237	12.855	2024.000	287.687	11.692	1.586
AMF plants	Mean	±SD	Mean	±SD	Mean	±SD
Gleysol	168.788	9.142	3282.400	841.324	19.372	4.608
Luvisol	172.471	10.899	3498.000	1254.965	20.099	6.753
Anthrosol	198.950	15.847	4089.800	627.357	20.717	3.916
Sterile peat	194.033	7.521	3018.400	298.686	15.585	1.760
Open field	Mean	±SD	Mean	±SD	Mean	±SD
Control plants	175.150	31.364	4105.200	1357.834	24.005	8.145
AMF plants	197.731	20.683	4419.800	1289.080	22.381	6.344

Table S2. The essential oil profile of the *Artemisia* plants under AMF treatment and without AMF (control) under semi-field (K1–control, G1-AMF treated) and open field conditions (K2–control, G2-AMF treated).

**Extraction**	**G1 (%, ml/100 g fresh leaf)**	**K1 (%)**	**G2 (%)**	**K2** (%)
1	1.104	0.576	1.25	0.427
2	0.999	0.527	1.049	0.392
3	1.051	0.553	1.149	0.409
Mean ± SD	1.052 ± 0.052	0.552 ±0.024	1.15 ±0.1	0.41 ±0.017

**Components**	**K1 (%)**	**G1 (%)**	**K2 (%)**	**G2 (%)**
Sabinene	0.39	0.40	0.58	0.34
β-pinene	4.50	5.45	5.50	3.17
β-myrcene	1.03	1.46	1.16	0.57
alpha-terpinene	0.26	0.27	0.29	0.00
p-cymene	1.03	1.06	2.17	0.79
Limonene	0.00	0.27	0.43	0.00
1.8-cineole	5.78	4.12	4.19	3.05
gamma-terpinene	0.64	0.66	0.72	0.57
cis-sabinene-hydrate	0.26	0.40	0.29	0.00
Terpinolene	0.13	0.13	0.14	0.00
trans-sabinene-hydrate	0.26	0.27	0.29	0.00
m-cymene	0.00	0.00	1.30	0.34
chrysanthenone	0.77	0.66	0.87	0.00
Camphor	33.55	35.08	32.11	26.92
Pinocarvone	1.16	1.33	1.16	0.90
Borneol	1.29	0.80	0.58	0.45
Pinocamphone	0.26	0.27	0.29	0.00

terpinen-4-ol	1.16	1.06	0.87	0.79
alpha-terpineol	0.39	0.27	0.43	0.00
Myrtenol	2.31	2.53	2.31	1.92
cis-carveol	0.64	0.53	0.87	0.00
D-carvone	0.26	0.27	0.14	0.00
Bornyl- acetate	0.13	0.13	0.29	0.00
Eugenol	0.51	0.40	0.29	0.00
alfa-cubebene	0.51	0.53	0.72	1.02
beta-caryophyllene	3.47	3.46	3.04	4.52
Humulene	0.26	0.27	0.43	0.00
beta-farnesene	8.10	8.38	7.52	16.85
germacrene D	10.67	11.57	12.00	23.52
alpha-guaiene	0.00	1.33	1.88	0.90
Elemene	1.29	1.33	1.45	2.38
Gurjunene	0.26	0.27	1.74	0.00
Spathulenol	1.16	1.06	1.01	2.04
caryophyllene-oxide	2.06	1.73	1.45	1.81
Copaene	1.41	1.46	1.59	0.90
alpha-bisabolol	5.14	4.79	2.89	1.47
Total	91.00	94.00	93.00	95.23
Monoterpene hydrocarbons (%)	12.98	13.63	15.87	8.40
Oxygenated monoterpenes (%)	38.60	40.73	37.39	29.51
Sesquiterpenes (%)	31.23	34.00	33.22	52.77
